# Van der Waals epitaxy and characterization of hexagonal boron nitride nanosheets on graphene

**DOI:** 10.1186/1556-276X-9-367

**Published:** 2014-07-28

**Authors:** Yangxi Song, Changrui Zhang, Bin Li, Guqiao Ding, Da Jiang, Haomin Wang, Xiaoming Xie

**Affiliations:** 1 State Key Laboratory of Advanced Ceramic Fibers and Composites, College of Aerospace Science and Engineering, National University of Defense Technology, 109 Deya Road, Changsha 410073, People's Republic of China; 2 State Key Laboratory of Functional Materials for Informatics, Shanghai Institute of Microsystem and Information Technology, Chinese Academy of Sciences, 865 Changning Road, Shanghai 200050, People's Republic of China

**Keywords:** Hexagonal boron nitride, Nanosheets, Graphene, van der Waals epitaxy, Chemical vapor deposition

## Abstract

Graphene is highly sensitive to environmental influences, and thus, it is worthwhile to deposit protective layers on graphene without impairing its excellent properties. Hexagonal boron nitride (h-BN), a well-known dielectric material, may afford the necessary protection. In this research, we demonstrated the van der Waals epitaxy of h-BN nanosheets on mechanically exfoliated graphene by chemical vapor deposition, using borazine as the precursor to h-BN. The h-BN nanosheets had a triangular morphology on a narrow graphene belt but a polygonal morphology on a larger graphene film. The h-BN nanosheets on graphene were highly crystalline, except for various in-plane lattice orientations. Interestingly, the h-BN nanosheets preferred to grow on graphene than on SiO_2_/Si under the chosen experimental conditions, and this selective growth spoke of potential promise for application to the preparation of graphene/h-BN superlattice structures fabricated on SiO_2_/Si.

## Background

Graphene has attracted global research interests across a wide range of applications [1,2]. However, graphene is highly sensitive to extraneous environmental influences. Thus, it was deemed worthwhile to deposit protective layers over graphene without impairing its properties. Hexagonal boron nitride (h-BN), a well-known dielectric material, may afford the necessary protection for graphene [3,4].

As an analogue of graphene, h-BN shows a minimal lattice mismatch with graphene of about 1.7%, yet has a wide band gap [5-8] and lower environmental sensitivity [3,4]. Hence, h-BN proves to be a promising dielectric material, or substrate, for two-dimensional electronic devices and especially for those based upon the use of graphene [9-13]. Graphene, partially covered by h-BN protective layers, may display promising electronic characteristics of graphene with much lower environmental sensitivity.

Recently, chemical vapor deposition (CVD) synthesis of h-BN on Ni [14-16] or Cu [13,17-19] substrates has been further investigated. For the following applications in graphene electronic devices, h-BN can be acquired by etching of the catalyst substrates and a transfer technique. Nevertheless, the transfer process brings inevitable contamination or even destruction, and it is difficult to determine the position and the coverage ratio of h-BN on graphene. Considering this problem, we pay attention to the catalyst-free CVD growth of h-BN on graphene, which promises direct application in graphene electronic devices and may obviate the need for a transfer process.

It has been demonstrated that van der Waals epitaxy by catalyst-free CVD can be a promising route for the growth of topological heterostructures [20-22]. Moreover, the surface of graphene is atomically flat and without dangling bonds, which makes graphene a promising template for the van der Waals epitaxy of other two-dimensional materials. Compounds with 1:1 B/N stoichiometry are often selected as h-BN precursors for CVD, and borazine (B_3_N_3_H_6_) could be a promising choice as it would produce BN and hydrogen, which are both environmentally friendly.

In this research, the van der Waals epitaxy of h-BN nanosheets on mechanically exfoliated graphene by catalyst-free low-pressure CVD, using borazine as the precursor to h-BN, was demonstrated. The h-BN nanosheets preferred to grow on graphene rather than on SiO_2_/Si and tended to exhibit a triangular morphology when grown on a narrow graphene belt. The h-BN nanosheets grown on graphene were highly crystalline, albeit with various in-plane lattice orientations.

## Methods

h-BN nanosheets were synthesized in a fused quartz tube with a diameter of 50 mm. Graphene was transferred onto silicon oxide/silicon (SiO_2_/Si) wafers by mechanical exfoliation from highly oriented pyrolytic graphite (HOPG, Alfa Asear, Ward Hill, MA, USA). The h-BN precursor (borazine) was synthesized by the reaction between NaBH_4_ and (NH_4_)_2_SO_4_ and purified according to our previous reports [23,24]. The temperature for the CVD growth of h-BN nanosheets was set to 900°C. Before the growth of h-BN, with the tube heated to 900°C, graphene grown on SiO_2_/Si was first annealed for 60 min in an argon/hydrogen flow (Ar/H_2_, 5:1 by volume, both gases were of 99.999% purity from Pujiang Co., Ltd, Shanghai, China) of 180 sccm to remove pollutants remaining on the graphene after mechanical exfoliation. During the growth process, borazine, in a homemade bubbler, was introduced to the growth chamber by another Ar flow of 2 sccm, while the Ar/H_2_ flow remained unchanged. The typical growth time was 5 min, while the pressure was 10 to 100 Pa. After the growth process, the tube was rapidly cooled to room temperature.

Raman spectroscopy was performed in a Thermo DXR with 532-nm laser excitation (Thermo Fisher Scientific, Waltham, MA, USA). Atomic force microscopy (AFM) (Dimension Icon, Bruker, Karlsruhe, Germany) and scanning electron microscopy (SEM) (Nova NanoSEM 320, FEI Co., Hillsboro, OR, USA) were used to observe the thickness and morphology of the h-BN nanosheets. X-ray photoelectron spectroscopy (XPS) (AXIS Ultra, Kratos Analytical, Ltd, Manchester, UK) was conducted to analyze the chemical composition of the films. The h-BN nanosheets with the graphene substrate were transferred to transmission electron microscopy (TEM) grids for further characterization. Both morphology images and selected area electron diffraction (SAED) patterns of the h-BN nanosheets were obtained by field emission high-resolution transmission electron microscopy (HRTEM) (Tecnai G^2^ 20, FEI Co.).

## Results and discussion

**Figure 1:**
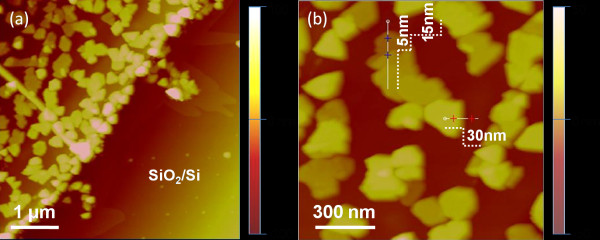
**AFM images of h-BN/graphene on SiO**_**2**_**/Si. (a)** Boundary region of h-BN/graphene and SiO_2_/Si. **(b)** h-BN nanosheets on graphene.

AFM images (Figure 1) show the morphology and thickness of the h-BN nanosheets. Figure 1a shows the boundary region of SiO_2_/Si and graphene with its associated h-BN nanosheets. Figure 1b displays the polygonal morphology of the h-BN nanosheets. It was interesting to note that h-BN nanosheets preferred to grow on graphene rather than on SiO_2_/Si.

This result possibly originated from the minimal lattice mismatch between h-BN and graphene, and the small amount of defects remaining in the graphene after mechanical exfoliation and high temperature annealing, and these would enable the h-BN to nucleate on graphene and grow thereafter. This selective growth phenomenon promises potential applications for graphene/h-BN superlattice structures fabricated on SiO_2_/Si.

**Figure 2:**
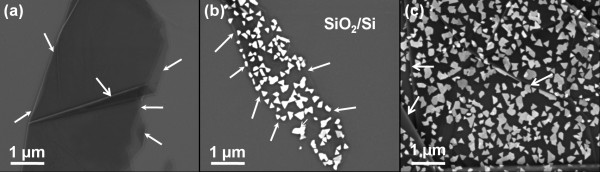
**SEM images of graphene and h-BN/graphene on SiO**_**2**_**/Si. (a)** Multilayer graphene on SiO_2_/Si before CVD, with the graphene boundary, and wrinkling, indicated by arrows. **(b)** h-BN nanosheets on a narrow graphene belt on SiO_2_/Si, with the graphene boundary indicated by arrows. **(c)** h-BN nanosheets on a larger graphene film, with wrinkles indicated by arrows.

This same phenomenon was also seen in SEM images as shown in Figure 2. Figure 2a shows graphene on SiO_2_/Si before CVD, while Figure 2b,c shows h-BN/graphene on SiO_2_/Si after CVD. It took time to distinguish graphene from SiO_2_/Si due to their low contrast under the SEM as shown in Figure 2a,b where the boundaries of graphene zones on the SiO_2_/Si substrate are indicated by arrows. The wrinkles in the graphene in Figure 2a,c originated from the mechanical exfoliation process and could also act as markers indicating the presence of graphene.

The h-BN nanosheets exhibited a polygonal morphology with some nanosheets becoming isolated islands on the graphene, while others with different thicknesses joined and became stacked, as shown in Figure 2c. Moreover, the h-BN nanosheets tended to exhibit a triangular morphology on the much narrower graphene belt, as shown in Figure 2b. This result is similar to van der Waals epitaxial growth of MoS_2_ on graphene [21] and perhaps originates from the higher boundary effect of the narrower graphene belt after mechanical exfoliation [25]. Besides, the triangular h-BN nanosheets on graphene showed different in-plane orientations from each other.

Raman spectroscopy provided a useful means of gleaning information about the lattice vibration modes of graphene and h-BN. After being transferred to SiO_2_/Si by the Scotch tape mechanical exfoliation method, the graphene was generally aligned with the (002) lattice plane parallel to the surface of the SiO_2_/Si wafer [1,2].

**Figure 3:**
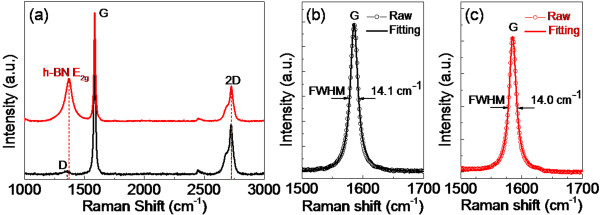
**Raman spectra. (a)** Raman spectra of graphene before CVD (lower plot) and h-BN/graphene after CVD (upper plot). G peaks fitting with Lorentz curves (solid lines) for graphene substrate **(b)** and h-BN/graphene **(c)** are shown with their FWHMs, respectively.

The existence of graphene was shown by Raman spectra in Figure 3, in which the *I*_2D_/*I*_G_ ratio of graphene was less than 0.5, indicating the multilayer structure of the graphene. Moreover, a weak D peak of graphene at 1,350 cm^-1^ was observed from the Raman spectra (Figure 3), indicating a small number of defects in the graphene, which may have originated from the original HOPG or the mechanical exfoliation process. For the sample examined after CVD, a peak much stronger than the D peak of graphene appeared at 1,367 cm^-1^, indicating the *E*_2g_ vibration mode of h-BN, which was consistent with the reported values [5,6,13-19]. Interestingly, the 2D and G peaks for graphene diminished in intensity after CVD, and this may have originated from the partial coverage of the graphene by h-BN. As shown in Figure 3b,c, the G peaks of graphene for the graphene substrate and h-BN/graphene were fitted with Lorentz curves (solid lines). The fitting data were well fitted with the raw data, while the Raman frequency and full width at half maximum (FWHMs) for G bands were almost equal to each other. These results are comparable with the reported values of graphene [26] and graphite [27,28], showing the high quality of graphene before and after CVD and indicating that the synthesis of h-BN nanosheets on graphene in our manuscript does not cause a degradation of graphene.

According to previous reports [29], the gas-phase nucleation for h-BN was absent at growth temperatures lower than 1,000°C; hence, the growth of h-BN nanosheets on graphene was dominated by the surface nucleation during our CVD process at 900°C. Moreover, the surface topography of the substrate is vital to the surface nucleation [30]. Consequently, the nucleation of the h-BN nanosheets on the graphene substrate was regulated by the surface morphology of graphene in our work. Additionally, the atomic scale defects, dislocations, and steps for the graphene substrate were inevitable during the mechanical exfoliation process due to the strong interlayer binding of graphite [31], and the atomic-level defects, dislocations, and steps of the substrates would serve as the nucleation centers for CVD growth, for the curved sp2 π bonds in the graphene defects, dislocations, and steps were more reactive than the planar graphene regions [21,32]. In our work, a small number of defects for the graphene substrates were proved by the weak D peak of Raman spectra in Figure 3. The atomic defects offer additional bond sites to the carbon atoms, making them energetically preferred for nucleation. During the CVD growth, the atomic-level defects of graphene could effectively cause nucleation of the h-BN on the graphene. Subsequently, with an increased amount of precursor, the h-BN nanosheets could grow on the surface of graphene through weak van der Waals interactions.

**Figure 4:**
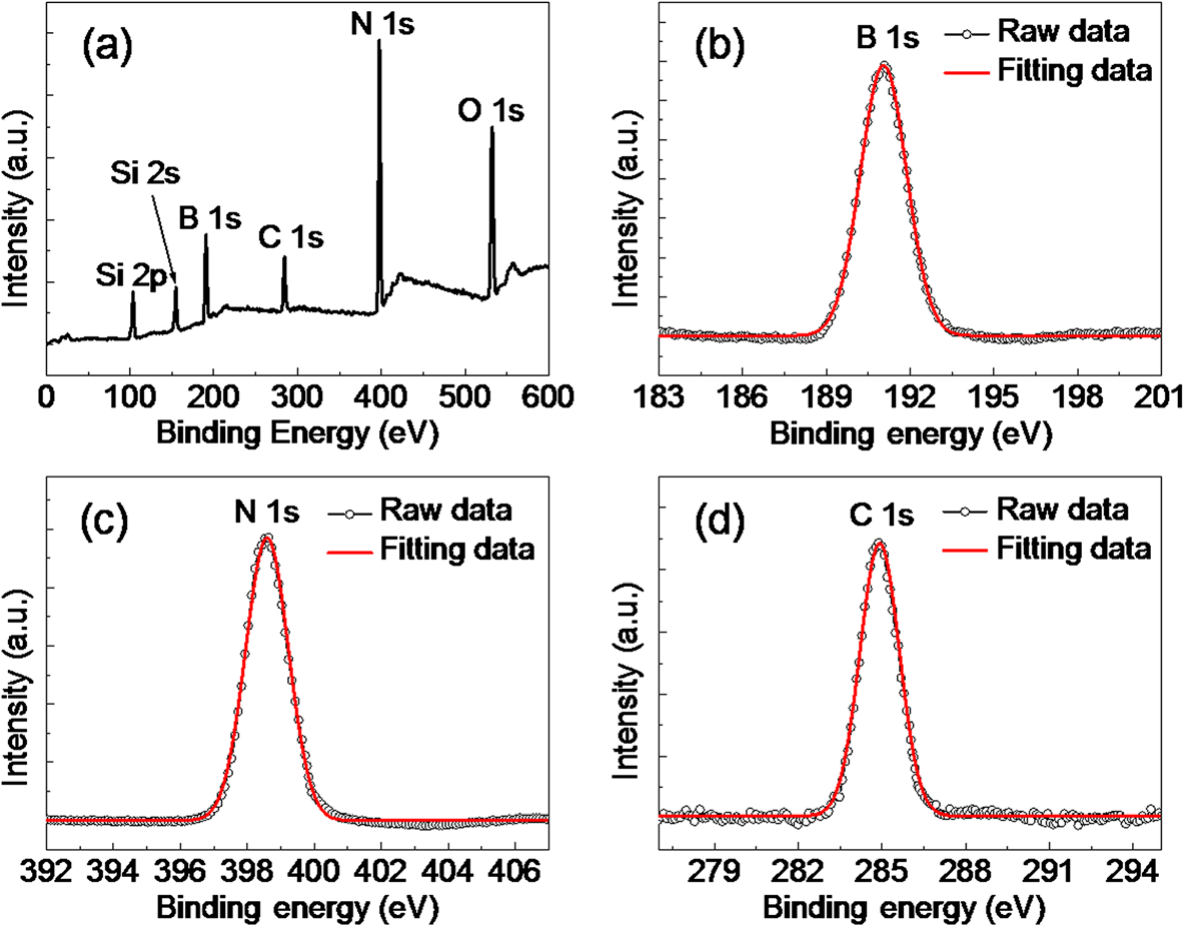
**XPS spectra of h-BN/graphene on SiO**_**2**_**/Si. (a)** Survey spectrum. **(b-d)** XPS spectra of B1s, N1s, and C1s core levels, respectively. The peaks of (b-d) were fitted with Gaussian curves (red peaks), and good fits could be observed for the raw data and the fitting data.

XPS was used to analyze the chemical composition of the h-BN/graphene on the surface of the SiO_2_/Si, as shown in Figure 4. The raw XPS data were corrected using the binding energy of the C-C bond at 284.5 eV. The Si and O peaks in Figure 4 arose from the SiO_2_/Si substrate, while the C peak arose from the presence of graphene. The binding energies of B1s and N1s from the XPS spectra were 191.0 and 398.5 eV, respectively, which were in good agreement with reported values [14,16,18,19,33,34] for h-BN. The B/N ratio of the sample, as taken from the XPS measurement, was 1.01, indicating the nearly stoichiometric composition of the synthesized h-BN nanosheets on graphene. As shown in Figure 4b,c,d, the XPS peaks of B1s, N1s, and C1s core levels were fitted with Gaussian curves (red peaks). The fitting data were well fitted with the raw data, and no shoulder peaks could be observed from the fitting curves. Hence, the single peaks of fitting data indicate that the C-B or C-N bonds do not exist in our h-BN/graphene system, compared with the reported results of BCN films [35,36]. These results show that the synthesis of h-BN nanosheets on graphene in our manuscript does not cause a degradation of graphene.

We have pointed out the reason for the nucleation of the h-BN on graphene. In fact, the deposition of h-BN nanosheets on graphene was performed as instantaneous nucleation followed by three-dimensional growth in our catalyst-free CVD growth. Similar results of three-dimensional growth in certain situations have been proved by previous reports [21,32]. As discussed above, energy optimization is of great importance to the nucleation of h-BN, and the defects, dislocations, and steps of graphene are energetically preferred. During the CVD growth of h-BN on graphene, the above energetically preferred regions of graphene would be covered or remedied by h-BN layers with a certain domain size. As an alternative, the edges of the as-grown h-BN layers and the regions near the defects of graphene turned energetically preferred for nucleation of new h-BN layers, which both favor the vertical or three-dimensional growth of h-BN nanosheets on the graphene.

**Figure 5:**
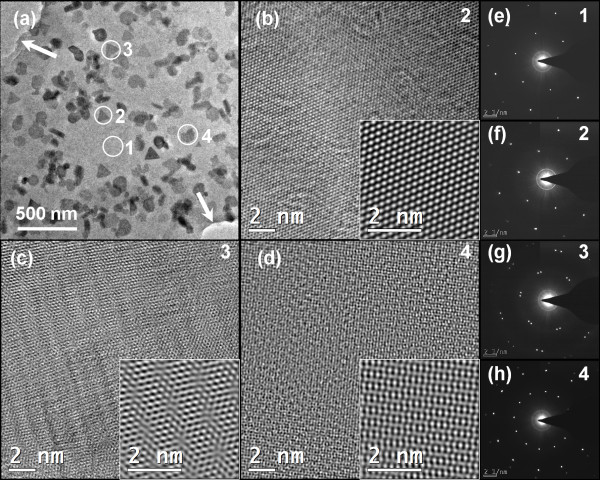
**Images of h-BN/graphene transferred onto TEM grids. (a)** A low-magnification TEM image of h-BN nanosheets on graphene, with the arrows showing the graphene boundary. **(b-d)** HRTEM atomic images corresponding to regions 2, 3, and 4 in (a), with the insets showing FFT-filtered images, respectively. **(e-h)** SAED patterns corresponding to regions 1 to 4.

After the h-BN nanosheets on graphene were transferred to TEM grids after the etching of SiO_2_/Si, atomic resolution HRTEM was used to study the crystalline structure of the aforementioned h-BN nanosheets on their respective graphene substrates. Figure 5a shows a TEM image of the h-BN nanosheets on graphene, with the arrows indicating the edge of the graphene. The polygonal objects on the graphene indicated the existence of h-BN nanosheets. The numbers ‘1’ to ‘4’ indicate typical regions of Figure 5a. Region 1 refers to a region of graphene without any h-BN nanosheet thereon, while regions 2 to 4 refer to isolated h-BN nanosheets on the graphene. Figure 5b,c,d shows the atomic images corresponding to regions 2 to 4, while the corresponding SAED patterns for regions 1 to 4 are shown in Figure 5e,f,g,h, respectively. The regular, periodic SAED spots evinced the high degree of crystallinity of both the graphene and h-BN nanosheets.Figure 5b shows that the h-BN nanosheet in region 2 had the same in-plane lattice orientation as the graphene substrate. However, the h-BN nanosheets and graphene in regions 3 and 4 were rotationally displaced, according to their Moiré patterns (see insets of Figure 5c,d, respectively). The h-BN nanosheets on graphene had various in-plane lattice orientations, which were consistent with the SAED patterns of Figure 5f,h. These results were also evinced by the SEM image (Figure 2b), as the triangular h-BN nanosheets on the narrow graphene belt also lay in various directions.

## Conclusions

In summary, we have demonstrated the van der Waals epitaxy of h-BN nanosheets on graphene by catalyst-free CVD, which may maintain the promising electronic characteristics of graphene. The h-BN nanosheets tended to have a triangular morphology on a narrow graphene belt, whereas they had a polygonal morphology on a much larger graphene film. The B/N ratio of the h-BN nanosheets on graphene was 1.01, indicative of an almost stoichiometric composition of h-BN. The h-BN nanosheets preferred to grow on graphene rather than on SiO_2_/Si, which offered the promise of potential applications for the preparation of graphene/h-BN superlattice structures. The h-BN nanosheets on graphene had a high degree of crystallinity, except for various in-plane lattice orientations. The synthesis of h-BN nanosheets on multilayer graphene has been studied, and h-BN nanosheets on few-layer and even monolayer graphene will be synthesized in future work. This may satisfy certain application requirements for topological heterostructures and graphene-related electronic devices.

## Competing interests

The authors declare that they have no competing interests.

## Authors' contributions

YS, CZ, BL, and XX designed the experiments, and YS carried out most of the experimental work and material characterizations. CZ and BL synthesized the borazine. YS, CZ, BL, GD, and XX discussed the results, and YS drafted the manuscript. All authors have read and approved the final manuscript.
